# Quality of Life in Patients Undergoing Hemodialysis or Peritoneal Dialysis Treatment

**DOI:** 10.4021/jocmr552w

**Published:** 2011-05-19

**Authors:** Paraskevi Theofilou

**Affiliations:** Department of Psychology, Panteion University, Eratous 12, 14568, Athens, Greece. Email: theofi@otenet.gr

## Abstract

**Background:**

“Does the type of dialysis treatment make a difference to the quality of life (QoL) and mental health of renal patients in Athens?” The study investigated the differences in 84 in-center hemodialysis (HD) and 60 continuous ambulatory peritoneal dialysis (CAPD/PD) patients.

**Methods:**

Patient-reported assessments included: WHOQOL-BREF inventory of World Health Organization, General Health Questionnaire (GHQ-28) of Goldberg, State-Trait Anxiety Inventory, Center for Epidemiologic Studies Depression Scale (CES-D) and Multidimensional Health Locus of Control (MHLC).

**Results:**

Results indicated that HD patients reported lower QoL in the environment and social relationships domains. More symptoms were also reported in the GHQ-28 subscales of anxiety/insomnia and severe depression. This measurement includes sleep problems and suicidal thoughts.

**Conclusions:**

The findings confirm the differences between the two treatment modalities, indicating that HD patients have poorer QoL in several aspects of their environment and their social relationships. Both groups reported elevated depression. However, HD patients reported more suicidal thoughts and sleep problems compared to PD patients.

**Keywords:**

Quality of life; Hemodialysis; Peritoneal dialysis; Renal disease

## Introduction

Modern societies include increasing proportions of elderly people, with a resulting increase in the incidence and duration of chronic illnesses. Similarly, advanced age is considered a significant determinant of depression [1] and poor quality of life (QoL) [2, 3]. Additionally, the provision of therapies relevant to chronic diseases addresses the issues beyond the concept of cure, bringing to the center the need for a dignified QoL of patients [4]. An increased interest in QoL is observed in patients who suffer from chronic diseases, including those with end-stage renal disease [5, 6].

Regarding patients either in hemodialysis (HD) or continuous ambulatory peritoneal dialysis (CAPD/PD) treatment modalities, the QoL differences reported in the relevant literature, are inconclusive [7]. Generally, studies that examined generic QoL, have indicated that although global QoL levels are comparable between the two treatment categories, there are treatment-related differences in dialysis-specific aspects of QoL, with some domains better for HD and others better for PD group. For example, HD patients have reported higher scores in measurements of physical well-being [8], including better sleep and sexual life [9]. Such evidence was seen in the first two years of dialysis and over a period of time [9-11]. However, findings are mixed. Sleeping problems and distress during the night, before the dialysis, have been indicated by HD patients [12-15]. On the other hand, compromised physical well-being in CAPD patients has been reported in connection with lower levels of albumin [11] and health complications, e.g., peritonitis [16].

Regarding mental health, HD are reported to present more depressive symptoms than PD patients [12, 17, 18]. Depression could be linked to the HD treatment requiring continuous connection with the hemodialysis machine and the patients experiencing serious restrictions on their level of independence [12, 17]. The rate of suicidal attempts in HD group is high, while a considerable number of deaths caused by dietary violations, may be also linked to suicide commitment [19, 20]. Furthermore, HD patients are reported to face psychosocial problems, e.g., conflictuous interactions with their medical carers. Such findings can be attributed to the stressful conditions in the HD treatment modality, with frequent visits and prolonged waiting time in the HD unit [12, 21, 22].

It appears that psychological indicators tend to favor PD patients. This can be due to the PD treatment offering increased autonomy and control, flexibility in everyday life and reduction of dietary and social restrictions [10, 23-25]. Specifically, PD group has indicated better QoL ratings in ‘perceived ability to travel’, ‘financial concerns’, ‘restriction in eating and drinking’ and ‘dialysis access problems’ [9, 26]. Further on, PD patients have indicated more positive ratings in several kidney-disease QoL domains, e.g., kidney disease burden and encouragement satisfaction with medical care [27].

In overall, end-stage renal disease patients have to cope with many adversities, like physical symptoms, special diet schedules, changes in their body image while the outcome of treatment is not standard [28]. They also have to reconsider their personal, social and professional goals within the context of living with chronic illness. End-stage renal disease is considered to have serious effects on the patients’ QoL and may affect negatively the social, financial and psychological aspects of their life [29-31].

The purpose of this study is to examine differences in QoL as well as mental health between HD and PD patients. We mainly hypothesize that HD patients present lower levels of QoL indicating more symptoms of depression and anxiety.

## Materials and Methods

Participants were recruited from three General Hospitals in Athens. The sample included 84 patients undergoing in-center hemodialysis (HD) and 60 undergoing continuous ambulatory peritoneal dialysis (CAPD). Most HD patients were male (a higher percentage of males is reported to suffer from chronic renal failure) [32], single, slightly younger than females and with less years of education.

The subjects (HD, PD) were selected according to the following criteria: 1) diagnosis of end-stage renal disease, 2) current HD or PD treatment, 3) age above 18, 4) native language-Greek, and 5) volunteer participation and signed consent form.

All subjects had been informed of their rights to refuse or discontinue participation in the study, according to the ethical standards of the Helsinki Declaration of 1983. Ethical permission for the study was obtained from the scientific committees of the hospitals.

### Measurement tools

#### World Health Organization Quality of Life instrument (WHOQOLBREF) [33]

It is a self-report generic QoL inventory of 26 items, validated within Greek populations [34]. The items fall into 4 domains: a) Physical health, b) Psychological well-being, c) Social Relationships, and d) Environment. Two of the items provide a facet measuring Overall QoL/health. It is rated on a 5-point Likert scale and the range of scores is between 1 and 20 with higher scores indicating better QoL.

#### General Health Questionnaire (GHQ-28)

It is a widely used self-report measure of general health, developed by Goldberg [35], and validated with Greek populations [36]. It may identify short-term changes in mental health and is often used as a screening instrument for psychiatric cases in medical setting and general practice. The 28-item version used in this study, consists of four sub-scales: a) somatic symptoms, b) anxiety/insomnia, c) social dysfunction, and d) severe depression. Higher scores indicate a worse general health status.

#### State-Trait Anxiety Inventory (STAI 1/STAI 2)

It consists of 20 items referring to self-reported state anxiety and 20 items to trait anxiety [37, 38]. State anxiety reflects a “transitory emotional state or condition of the human organism that is characterized by subjective, consciously perceived feelings of tension and apprehension, and heightened autonomic nervous system activity”; it may fluctuate over time and can vary in intensity. In contrast, trait anxiety denotes “relatively stable individual differences in anxiety proneness” and refers to a general tendency to respond with anxiety to perceived threats in the environment [37]. Higher scores mean that patients are more anxious.

#### Center for Epidemiologic Studies Depression Scale (CES-D) [39-41]

It is a 20-item self-report measure of depression. A higher score means that the patient is more depressed. A value above 9.03 is required for a subject to be classified as depressed [41].

#### Multidimensional Health Locus of Control (MHLC)

It is a self-report tool measuring health behavior in terms of internal beliefs about the current condition of health. The inventory consists of 18 items, which comprise 4 categories of beliefs: a) internal locus, b) chance, c) doctors, and d) important others. The last three refer to external locus of control [42, 43]. The 4 categories are not mutually exclusive and scores may weight in a particular direction. Higher scores indicate stronger presence of the specific dimension of beliefs.

## Results

**Table 1:** Sociodemographic Characteristics of the Sample (N = 144)

	HD N = 84 (58.3%)	PD N = 60 (41.7%)
Age (years) Mean (SD)	58.12 (16.11)	64.28 (12.51)
Gender		
Male	55 (65.5%)	31 (51.7%)
Female	29 (34.5%)	29 (48.3%)
Total	84 (100.0%)	60 (100.0%)
Marital status		
Single	19 (22.6%)	6 (10.0%)
Married	58 (69.0%)	49 (81.7%)
Divorced	1 (1.2%)	0 (0%)
Widowed	6 (7.1%)	4 (6.7%)
Roommate	0 (0%)	1 (1.7%)
Total	84 (100.0%)	60 (100.0%)
Education		
Elementary	42 (50.0%)	20 (33.3%)
Secondary	26 (31.0%)	30 (50.0%)
University	16 (19.0%)	10 (16.7%)
Total	84 (100.0%)	60 (100.0%)

The total sample of 144 patients consisted of 86 males (59.7%) and 58 females (40.3%), with a mean age of 60.6 years. Descriptive statistics are presented in Table 1. HD patients were receiving current dialysis treatment for a mean duration of 7.3 ± 7.1 years, while PD patients for 3.2 ± 2.0 years. There were 9 missing values due to information missing from the patients medical records.

**Table 2:** Mean Scores ± SD of WHOQOL-BREF Domains and the Overall QoL/Health Facet (Independent-Samples T Test Demonstrating Differences Between HD and PD Patients)

WHOQOL-BREF domains	HD Patients (N = 84)	PD Patients (N = 60)	P-value
	Mean ± SD	Mean ± SD	
Physical	12.71 ± 3.70	13.70 ± 2.96	0.08
Psychological	13.26 ± 3.65	13.36 ± 3.14	0.86
Social relationships	12.89 ± 3.51	14.03 ± 2.43	0.02*
Environment	13.00 ± 2.71	14.52 ± 1.78	0.00*
Overall QoL/health	3.00 ± 1.07	3.15 ± 0.82	0.34

* P < 0.05; N = 144

The values of the two groups were found to pass the normality distribution test. HD indicated lower QoL scores in the domains of environment and social relationships (P < 0.05) (Table 2). Further, they reported higher scores in the GHQ-28 sub-scales of anxiety/insomnia (P < 0.05), severe depression (P < 0.05), and in the total GHQ-28 score (Table 3).

**Table 3:** Mean Scores ± SD of GHQ-28 Health Subscales (Independent-Samples T Test Showing Differences Between HD and PD Patients)

GHQ-28 subscales	HD Patients (N = 84)	PD Patients (N = 60)	P-value
	Mean ± SD	Mean ± SD	
Somatic symptoms	1.86 ± 0.55	1.69 ± 0.52	0.06
Anxiety/insomnia	1.88 ± 0.65	1.48 ± 0.58	0.00*
Social dysfunction	2.30 ± 0.51	2.21 ± 0.40	0.25
Severe depression	1.58 ± 0.77	1.30 ± 0.56	0.02*
Total score	1.90 ± 0.50	1.67 ± 0.44	0.01*

* P < 0.05; N = 144

However, no statistically significant differences were found between the two groups in the independent scales of depression with CES-D and anxiety with STAI 1 & 2 (Table 4). It is noteworthy that following further analysis in order to detect depression in individual cases (with the use of cut-off points presented earlier in the instruments section), both HD and PD have values that are above the suggested level and are thus considered depressed (M = 12.43 and 13.26) [41].

**Table 4:** Mean Scores ± SD of Depression and State-Trait Anxiety (Independent-Samples T Test Showing Differences Between HD and PD Patients)

CES-D	HD Patients (N = 84)	PD Patients (N = 60)	P-value
	Mean ± SD	Mean ± SD	
Depression	12.43 ± 13.63	13.26 ± 10.40	0.74
STAI 1			
State Anxiety	29.68 ± 11.12	30.63 ± 8.92	0.66
STAI 2			
Trait Anxiety	35.15 ± 8.96	35.50 ± 9.35	0.87

N = 144

As far as internal beliefs, both groups presented similar patterns, with internal being the major locus of control, followed by chance, then by doctors and last by important others. However, some differences were also identified: HD presented comparatively higher scores in the dimension of internal locus, which can be indicative of a relatively stronger belief of their own control over their current condition of health. On the other hand, PD presented higher scores in the dimension of doctors, perhaps indicating a comparatively stronger belief of the control of their medical carers over their current condition of health and treatment (Table 5).

**Table 5:** Mean Scores ± SD of Health Locus of Control Factors (Independent-Samples T Test Showing Differences Between HD and PD Patients)

Health Locus of Control factors	HD Patients (N = 84)	PD Patients (N = 60)	P-value
	Mean ± SD	Mean ± SD	
Internal locus	26.89 ± 6.85	24.17 ± 8.11	0.03*
Chance	24.62 ± 8.22	22.84 ± 9.06	0.23
Doctors	16.00 ± 2.59	17.01 ± 1.61	0.00*
Important others	12.39 ± 4.44	12.19 ± 4.70	0.79

* P < 0.05; N = 144

## Discussion

The results of the study provide support regarding expected QoL and mental health differences between the two treatment-modality groups. Patients in the HD treatment, compared to PD treatment patients, reported a more compromised QoL in the domains of environment and social relationships. Accordingly, the HD group indicated experiencing less support from their community and social relationships. This may be associated with the restrictions imposed on their lives and the dependence on the dialysis procedure, which may cause social isolation [15, 26]. Also, it may be associated with the continuous stressful conditions governing HD treatment, e.g., high frequency of received in-center treatment and prolonged waiting-time in the medical unit [12, 21, 22].

Furthermore, HD compared to PD patients, indicated more problems in different aspects of their environment with a more negative evaluation, including availability/quality of health services, transportation, finances, recreation and opportunities for acquiring new skills and knowledge.

Regarding mental health, HD patients were found to evaluate less favorably their overall health status, reporting more physical and psychological symptoms, such as anxiety, sleeping problems and suicidal thoughts. It is noteworthy that both groups were considered depressed according to their values in the CES-D scale [41]. The higher levels of depression and anxiety in the GHQ sub-scales were not observed in the independent scales of CES-D and STAI 1. A possible explanation may be that different aspects of depression are measured by different instruments. Subsequently, the GHQ severe depression subscale includes items about suicidal thoughts, which are not measured by the CES-D scale. The comparatively higher scoring of HD patients on a scale that examines suicidal thoughts is in agreement with higher rates of suicidal attempts reported in this group [18-20].

Similarly, the GHQ anxiety/insomnia and STA1 scales do not measure the same content (e.g., items on sleep problems are not included in the latter scale).

Findings in the relevant literature regarding self-reported physical and psychological well-being, are mixed. In several studies, HD reported better scores than PD on physical well-being [8]. Similarly, prospective studies have shown a more favourable effect of HD treatment on physical QoL over time [9].

Regarding internal beliefs about current condition of health, both groups are comparable presenting a similar pattern, that is scoring higher in the dimension of internal locus, which is followed by the dimensions of chance, doctors and important others. A difference between the two treatment modalities concerns the relatively higher values of the HD patients in internal locus, indicating probably the emphasis on their own behavior for control over their current condition of health, perhaps a counterbalance for feelings of dependence on the machine. On the contrary, PD seemed to give more importance to the function of doctors, probably because they need to be trained in peritoneal dialysis procedures with the help of medical professionals. These findings may have also a relevance to the previously mentioned higher level of state anxiety in early starters of PD treatment.

In overall, there is evidence supporting that patients in HD treatment were in a more compromised position in comparison to PD. Social relationships and perceptions of environment, including health services, seemed to suffer. In terms of mental health, HD patients reported more anxiety/insomnia, depressive and suicidal symptoms. Regarding locus of control about current condition of health, both groups seemed to follow comparable patterns, while a difference was observed over the weight of each group on internal and external control.

Regarding limitations, patients were recruited from three different renal units and were a convenience sample. Thus, it was not possible for the two patient groups to be well-matched for demographic variables. Also, patients varied with respect to the length of current treatment, and the analysis of differences for this variable was performed post-hoc. Further, due to missing information in the patients records, there were some missing values for the latter variable. The data also of this study are cross-sectional and therefore do not provide insight into the longitudinal effects of current dialysis treatment on QoL and mental health. Finally, evidence provided by the results of this study can be extended by the control of the above issues and the use of even larger samples.
